# Transcription-Associated R-Loop Formation across the Human *FMR1* CGG-Repeat Region

**DOI:** 10.1371/journal.pgen.1004294

**Published:** 2014-04-17

**Authors:** Erick W. Loomis, Lionel A. Sanz, Frédéric Chédin, Paul J. Hagerman

**Affiliations:** 1 Department of Biochemistry and Molecular Medicine, University of California, Davis, School of Medicine, Davis, California, United States of America; 2 Department of Molecular and Cellular Biology, University of California, Davis, Davis, California, United States of America; 3 The Genome Center, University of California, Davis, Davis, California, United States of America; 4 MIND Institute, University of California, Davis, Health System, Sacramento, California, United States of America; CABIMER, Universidad de Sevilla, Spain

## Abstract

Expansion of a trinucleotide (CGG) repeat element within the 5′ untranslated region (5′UTR) of the human *FMR1* gene is responsible for a number of heritable disorders operating through distinct pathogenic mechanisms: gene silencing for fragile X syndrome (>200 CGG) and RNA toxic gain-of-function for FXTAS (∼55–200 CGG). Existing models have focused almost exclusively on post-transcriptional mechanisms, but co-transcriptional processes could also contribute to the molecular dysfunction of *FMR1*. We have observed that transcription through the GC-rich *FMR1* 5′UTR region favors R-loop formation, with the nascent (G-rich) RNA forming a stable RNA:DNA hybrid with the template DNA strand, thereby displacing the non-template DNA strand. Using DNA:RNA (hybrid) immunoprecipitation (DRIP) of genomic DNA from cultured human dermal fibroblasts with both normal (∼30 CGG repeats) and premutation (55<CGG<200 repeats) alleles, we provide evidence for *FMR1* R-loop formation in human genomic DNA. Using a doxycycline (DOX)-inducible episomal system in which both the CGG-repeat and transcription frequency can be varied, we further show that R-loop formation increases with higher expression levels. Finally, non-denaturing bisulfite mapping of the displaced single-stranded DNA confirmed R-loop formation at the endogenous *FMR1* locus and further indicated that R-loops formed over CGG repeats may be prone to structural complexities, including hairpin formation, not commonly associated with other R-loops. These observations introduce a new molecular feature of the *FMR1* gene that is directly affected by CGG-repeat expansion and is likely to be involved in the associated cellular dysfunction.

## Author Summary

Expansion of a CGG-repeat element within the human *FMR1* gene is responsible for multiple human diseases, including fragile X syndrome and fragile X-associated tremor/ataxia syndrome (FXTAS). These diseases occur in separate ranges of repeat length and are characterized by profoundly different molecular mechanisms. Fragile X syndrome results from *FMR1* gene silencing, whereas FXTAS is associated with an increase in transcription and toxicity of the CGG-repeat-containing mRNA. This study introduces a previously unknown molecular feature of the *FMR1* locus, namely the co-transcriptional formation of three-stranded R-loop structures upon re-annealing of the nascent *FMR1* transcript to the template DNA strand. R-loops are involved in the normal function of human CpG island promoters in that they contribute to protecting these sequences from DNA methylation. However, excessive R-loop formation can lead to activation of the DNA damage response and result in genomic instability. We used antibody recognition and chemical single-stranded DNA footprinting to show that R-loops form at the *FMR1* locus with increasing frequency and greater structural complexity as the CGG-repeat length increases. This discovery provides a missing piece of both the complex *FMR1* molecular puzzle and the diseases resulting from CGG-repeat expansion.

## Introduction

The human fragile X mental retardation 1 gene (*FMR1*; HGNC:3775) contains a (CGG)_n_ trinucleotide repeat that is responsible for a family of heritable disorders affecting both early neurodevelopment (fragile X syndrome; FXS) and late-onset neurodegeneration (fragile X-associated tremor/ataxia syndrome; FXTAS) [1]–[4]. The repeat element is located in the 5′ untranslated region (5′UTR) of the gene, and is thus transcribed into mRNA but not translated into the amino acid sequence of the gene product, the *FMR1* protein (FMRP).

Alleles in the ∼55–200 CGG-repeat range are historically referred to as “premutation” alleles in reference to increased instability and the tendency in maternal transmission to expand into the “full mutation” range of FXS (>200 CGG repeats) [3], [5], [6]. Premutation alleles are also variably associated with several clinical phenotypes; in addition to FXTAS, these phenotypes include primary ovarian insufficiency (FXPOI) [7] and neurodevelopmental involvement [8], [9]. Contrary to the gene silencing observed in FXS alleles, premutation alleles are associated with increased transcriptional activity. Indeed, *FMR1* mRNA levels are positively correlated with size of the repeat expansion in the premutation range [10]. The molecular pathogenesis of the premutation disorders is generally considered to be a toxic RNA gain-of-function resulting from the expanded CGG-repeat region in the mRNA, but a definitive mechanism for the RNA involvement has not yet emerged [1], [11]–[15].

Stable RNA:DNA hybrids can form upon transcription of cytosine-rich template sequences because a guanine-rich RNA:cytosine-rich DNA heteroduplex is thermodynamically more stable than the corresponding DNA:DNA duplex [16], [17]. Recent work has revealed that such structures form throughout the human genome, particularly at CpG island promoters [18], [19]. Additionally, *in vitro* transcription experiments showed that CGG trinucleotide repeats alone are able to form R-loops [20].

R-loops at CpG island promoters serve a natural and important role in protecting CpG-rich regions from acquiring DNA methylation and becoming epigenetically silenced [18]. In addition, R-loop formation at the 3′ end of numerous human genes is thought to permit efficient transcription termination [19], [21]. However, R-loop formation has also been linked to genomic instability in numerous systems [22]–[24] and is thought to trigger recombination at class-switch regions [25], [26]. Recent results suggest that defects in mRNA processing can result in an R-loop-dependent activation of the DNA damage response, and to the accumulation of γH2AX, a histone variant associated with the repair of DNA breaks [27], [28]. R-loops at the Prader-Willi syndrome *Snord116* locus are responsible for chromatin decondensation and for regulating the transcription of nearby imprinted genes [29]. Thus, it appears that R-loop formation in the genome is a widespread, dynamic process that is sensitive to perturbation, and has both physiological roles and potential “toxic” consequences through activation of the DNA damage response.

Herein we present evidence for R-loop formation at the endogenous human *FMR1* locus, and explore the impact of CGG-repeat expansion and transcription induction on the extent of *FMR1* R-loop formation.

## Results

### *FMR1* 5′UTR Sequence Composition Predicts R-Loop Formation

**Figure 1:**
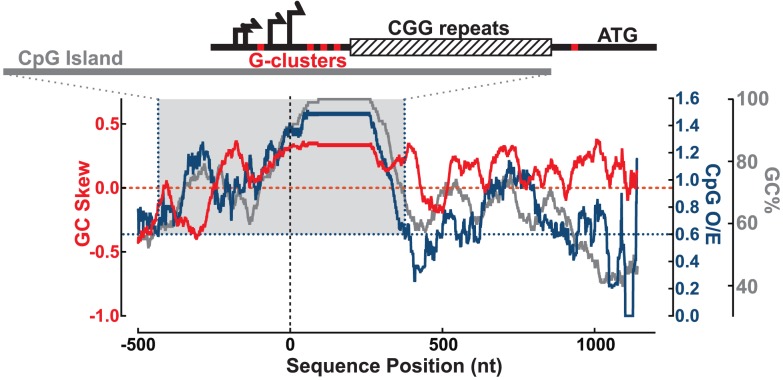
Sequence analysis of the *FMR1* promoter reveals signatures of R-loop formation. GC skew (red, left y-axis), CpG observed/expected ratio (CpG O/E; navy, right y-axis), and GC% (gray, right y-axis) calculated over a sliding 100 nt window from −500 to +1200 nt around the downstream-most known transcription start site (vertical dotted line). Gray-shaded box highlights CGI defined by CpG O/E>0.6 (navy dotted lines) and GC%>50% for at least 200 nt. Schematic at the top shows the *FMR1* 5′UTR with multiple transcription start sites (black arrows), G-clusters (red ticks), and CGG repeats (striped box), all overlapping the CGI (gray bar) for scale to the graph below.

We examined the sequence of the human *FMR1* 5′UTR to identify important features for R-loop formation, including proximity to transcription start sites (TSSs), GC skew, and G-clusters [18], [30]. We calculated the GC content (GC%) together with the density in CpG dinucleotides (CpG observed/expected ratio; CpG O/E) and GC skew (G−C/G+C) across the 5′ end of the *FMR1* gene (hg19 chrX:146,992,969–146,994,458; shown here for CGG = 100) (Figure 1). The multiple *FMR1* TSSs are located upstream of the CGG repeats and constitute the upstream boundary of the UTR [31]–[33], as depicted in Figure 1. Overall, the promoter and 5′UTR are exceptionally GC-rich, with GC% peaking at 100% through the repeats, and staying above 60% through the entire UTR (Figure 1). Part of this region also shows an elevated frequency of CpG dinucleotides and can be classified as a CpG island (CGI). The *FMR1* CGI overlaps with the promoter sequences and the 5′UTR through the CGG repeats (Figure 1), and therefore belongs to a large class of promoter CGIs [18]. It is notable that CGG-repeat expansions characteristic of FXTAS and FXS directly stretch out the 3′ boundary of the CGI promoter element, as defined by its high GC content and CpG density [34].

In addition to elevated GC% and CpG O/E, the *FMR1* CGI is also characterized by elevated GC skew downstream of the TSSs and through the CGG repeats (Figure 1). As noted for CGI promoters and other regions in the human genome, GC skew is highly predictive of R-loop formation [18], [19]. As with GC skew, G-clusters (≥4 Gs in a row) act as nucleation points for RNA:DNA hybridization [30]. Five such clusters are found in the 5′UTR, as indicated by red ticks on the schematic in Figure 1, including one that is included/excluded in the transcript depending on TSS choice. In total, these features predict R-loop formation at *FMR1* following transcription.

### DNA:RNA Immunoprecipitation Indicates the Formation of Genomic *FMR1* R-Loops

We used DNA:RNA immunoprecipitation (DRIP) to directly test the existence of R-loops at the endogenous *FMR1* locus in human genomic DNA, and compared the relative abundance of R-loops across the range of transcribed CGG-repeat expansion alleles. The S9.6 antibody recognizes RNA:DNA hybrids without any known sequence preference or sensitivity to DNA methylation ([18], [21], [35]; unpublished data). After DRIP, we calculated the fold enrichment of *FMR1* relative to input genomic DNA, and to a non-R-loop-forming genomic locus (*ZNF554*; HGNC:26629) using qPCR, where DRIP enrichment is not expected.

**Figure 2:**
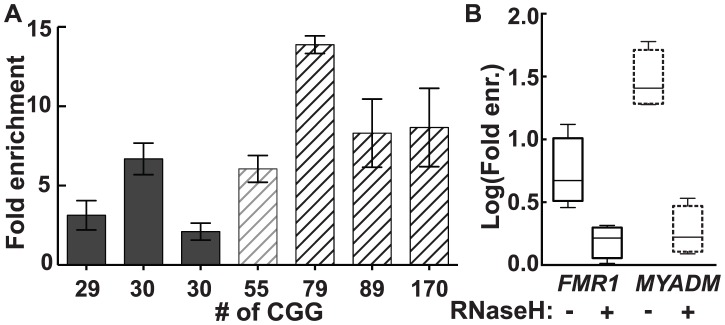
R-loop pull-down in human dermal fibroblasts confirms R-loop formation in the genome. (*A*) Fold enrichment for *FMR1* in dermal fibroblast cells cultured from seven individuals using a monoclonal antibody specific to hybrids. Enrichment is relative to input and a non-R-loop-forming genomic reference locus. (*B*) Treatment with recombinant RNases H1 and H2 (RNase H) eliminates enrichment seen for *FMR1* (solid lines) and *MYADM* (broken lines).

In genomic DNA from cultured human male dermal fibroblasts, we observed a 2.1- to 13.9-fold enrichment for *FMR1* across the range of CGG-repeat alleles tested (Figure 2*A*). Although there was substantial inter-subject variation in fold enrichment, both in control and premutation groups, the premutation group as a whole demonstrated greater enrichment (mean 9.0, SD 3.9, range 2.9–13.4) than the control group (mean 4.2, SD 2.4, range 1.6–8.6) (*P* = 0.0008; linear mixed-effects model, see: Material and Methods). By contrast, a positive control for a strong R-loop-forming locus, *MYADM* (HGNC:7544), showed consistently high enrichment (25- to 50-fold), which was not influenced by *FMR1* CGG-repeat size (Figure S1). As expected for R-loop formation, treatment with purified recombinant human RNases H1 and H2 eliminated DRIP pulldown. Hence, enrichment for *FMR1* in 3 different fibroblast lines went from a mean of 6.36±2.31 (SEM, n = 4) to 1.59±0.219 (SEM, n = 4) upon RNase H treatment (Figure 2*B*), a significant reduction (unpaired t-test on log-transformed enrichment values, *P* = 0.0125). Likewise, elimination of DRIP enrichment following RNase H treatment was also observed at the positive *MYADM* locus (unpaired t-test on log-transformed enrichment values, *P* = 0.0002) (Figure 2*B*).

### DOX-Induced Transcription and Expanded CGG Repeats Result in Enhanced *FMR1* R-Loop Formation

**Figure 3:**
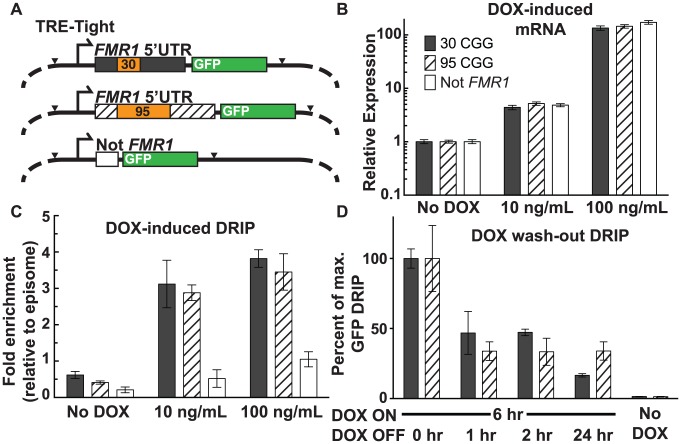
Effect of transcription and repeat length on *FMR1* R-loop formation. (*A*) Schematic of DOX-ON constructs with short or expanded *FMR1* CGG repeats or non-*FMR1* sequence, each with GFP reporter tags. Black arrowheads mark sites of restriction enzyme cleavage prior to DRIP, with EcoRI cutting at the start of the *FMR1* 5′UTR and XbaI cutting at the end of *EGFP*. (*B*) mRNA expression relative to non-induced cells for each construct. Error bars: SEM from 2 biological replicates. (*C*) DRIP fold enrichment of GFP fragment relative to the episome backbone. Error bars: SEM from 3 biological replicates. (*D*) DRIP percentage of input normalized to peak recovery (6 hours DOX ON) of GFP fragment at 0, 1, 2, and 24 hours post DOX washout, and No-DOX treatment. Error bars: SEM from 3 biological replicates.

We used a doxycycline (DOX)-inducible episomal system in SK-N-MC neuroepithelioma cells [36] to investigate the relationship between the frequency of transcription initiation and R-loop formation. The TRE-Tight promoter allows for precise control of transcription through an *FMR1* 5′UTR sequence harboring either a 95 or 30 CGG-repeat element, or a non-*FMR1* linker sequence ([36]; Figure 3*A*). All three constructs include *EGFP* cDNA, which was used as a target for qPCR to avoid amplification from endogenous sequences. Treatment with DOX at 10 ng/mL and 100 ng/mL resulted in a clear induction of transcription with equal expression levels for all three constructs, relative to the no-DOX baseline (Figure 3*B*).

Using DRIP-qPCR, we observed increased R-loop formation through the *FMR1* 5′UTR, mirroring the transcriptional response to DOX induction (Figure 3*C*; Figure S2 *A*). Fold enrichment for the 30 CGG-repeat allele increased from 0.62±0.096 (n = 3) without DOX, to 3.1±0.65 (n = 3) at 10 ng/mL DOX, and 3.8±0.24 (n = 3) at 100 ng/mL DOX. The 95 CGG-repeat allele increased from 0.41±0.044 (SEM, n = 3) without DOX, to 2.9±0.22 (n = 3) at 10 ng/mL DOX, and 3.45±0.50 (n = 3) at 100 ng/mL DOX. By contrast, the non-*FMR1* control locus showed little to no increase upon induction (Fig. 3*C*). We note that the episome backbone also showed modestly increased pull-down efficiency with increasing expression (Figure S2 *B*), which could result from R-loop formation around the *EGFP* poly(A) sequence. Indeed, R-loops have the propensity to form broad peaks around poly(A)-dependent termination regions ([19], [22]; F.C and L.S., unpublished observations). Given that R-loops inhibit the activity of restriction enzymes, this inhibition could prevent the cleavage required for separating the GFP restriction fragment from the background fragment and lead to apparent DOX-inducible R-loop formation over the episomal backbone. To account for this background, R-loop formation at the target GFP locus, as measured by DRIP-qPCR, was calculated relative to R-loop formation over the episome backbone and normalized to non-induced baseline (Figure 3*C*).

To assess the stability of R-loops once formed, we induced transcription for 6 hours with 100 ng/mL DOX, after which DOX was removed from the media and R-loop presence was measured by DRIP 1, 2, and 24 hours following the washout. Recovery of the 30-repeat allele decreased to 46.8%±15.3% (n = 2) of maximum after a 1-hour washout. It persisted at 47.2%±2.3% (n = 3) after a 2-hour washout, and dropped to 16.6%±1.2% (n = 3) after 24 hours (Figure 3*D*). Recovery of the 95-repeat allele decreased to 33.8%±6.7% (n = 2) of maximum after 1 hour, then remained essentially unchanged to 24 hours (33.9%±6.6%; n = 3) (Figure 3*D*). These data show that R-loop formation through the *FMR1* 5′UTR depends on active transcription initiation and that R-loops are dynamic structures, which are progressively formed and resolved.

### Mapping the ssDNA Structure of the *FMR1* Genomic R-Loop

**Figure 4:**
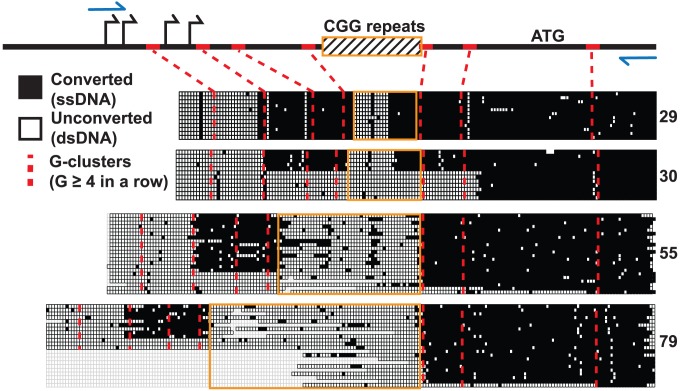
Non-denaturing bisulfite footprinting of the displaced DNA strand of the *FMR1* R-loop. Each row represents an individual sequence clone, grouped together for each allele size, from cultured human dermal fibroblasts. Each column is a cytosine position, with filled boxes representing converted, single-stranded DNA and open boxes representing unconverted, double-stranded DNA. Empty boxes represent sequence gaps from bacterial deletion or loss of clean sequencing signal. Schematic diagram at the top represents the *FMR1* 5′UTR with marked TSSs (black arrows), translation start (ATG), CGG repeats (striped box with orange border), PCR primers (blue arrows), and G-clusters (red ticks; red dotted lines).

We used non-denaturing sodium bisulfite treatment to map the extent of the displaced single-stranded DNA (ssDNA) constituting the *FMR1* R-loop in human male fibroblast genomic DNA. Sodium bisulfite deaminates unmethylated cytosines, but only with high efficiency in ssDNA. When applied in a non-denaturing manner, it can therefore be used as an efficient probe for R-loop formation and has been used extensively to footprint R-loop structures at single-nucleotide resolution [18], [26]. As expected from DRIP data, non-denaturing bisulfite footprinting revealed extensive single-strandedness through the 5′UTR across the range of transcribed *FMR1* alleles (CGG = 29, 30, 55, 79) (Figure 4). R-loop structures began at the first G-cluster downstream of the TSSs and continued through the repeat region for all four alleles examined. However, unlike any other region analyzed to date, stretches of unconverted cytosines indicative of double-stranded DNA were found inside the repeats. Furthermore, the extent of unconverted DNA was much greater for expanded alleles with higher CGG-repeat sizes (Figure 4). For normal CGG-repeat sizes, patterns of non-conversion were short and symmetrical and were centered on an AGG-repeat interruption not shown in the figure. The single converted CpG dinucleotide in the center of the unconverted CGG track of both 29- and 30-repeat samples was located immediately adjacent to the AGG polymorphism. Such a pattern would be expected if a short hairpin formed within the repeat region, with the stem of the structure being double-stranded and protected from conversion while a short loop is exposed. For longer CGG repeats, the footprinting data suggests that a large region of ssDNA exists upstream and downstream of the CGG repeat, but that most of the repeat region itself is in fact protected from conversion, save a few scattered points of conversion (Figure 4). Note that, for a certain number of molecules in the 30-, 55-, and 79-CGG samples, R-loops seemed to initiate at a G-cluster downstream of the repeats. Overall, these data show that R-loop formation at *FMR1* can initiate from different G-cluster seeding points, both upstream and downstream of the CGGs, and that R-loop formation through expanded CGG repeats may result in hairpin formation or other structural conformations.

## Discussion

At the DNA sequence level, R-loop formation is best predicted by the combination of GC content and GC skew, which measures the density and strand asymmetry in the distribution of guanines and cytosines, and correlates with the stability of RNA:DNA hybrids. In comparison to other R-loop forming regions of the genome, the normal (unexpanded) *FMR1* promoter matches “Class II” CGI promoters [18], [19]. This category is typical of skewed promoters on the X-chromosome and associates with marginally weaker GC skew [19]. As far as GC sequence composition is concerned, the human *FMR1* CGI is at the extreme end of the spectrum in the genome. A GC content of greater than ∼65% is generally viewed as “high GC,” and only 22 CGIs are listed at >80% in the human reference genome [37]. Our analysis shows that GC% at the *FMR1* promoter/5′UTR region peaks at 100% across the CGG-repeat, saturating this sequence characteristic. All three metrics of CGI composition and predictors of R-loop formation (GC%, CpG O/E, GC skew) peak at the repeat region. Importantly, CGG-repeat expansions associated with FXTAS and FXS will push *FMR1* into the category of stronger skewed promoters by increasing the lengths of the GC content, GC skew, and CpG tracks. CGG expansions are also likely to increase R-loop formation efficiency in two additional ways. First, expanded (premutation) alleles trigger higher transcriptional rates [10], which should favor the frequency of co-transcriptional R-loops. Second, expanded alleles are characterized by a shift in the usage of transcription initiation sites to upstream sites [31], [32]. This shift is expected to allow the inclusion of additional G-clusters, which are *de facto* R-loop initiation points, in the transcript. The CGG-repeat expansions that associate with FXTAS and FXS are therefore unique in that they strongly enhance the propensity of the *FMR1* CGI to form co-transcriptional R-loops.

Here, we provide direct experimental evidence that R-loops form at the endogenous genomic *FMR1* locus, which was first detected by the ability of the S9.6 anti-RNA:DNA hybrid antibody to specifically immunoprecipitate the *FMR1* locus. As expected, enrichment was lost following enzymatic resolution of the RNA:DNA hybrids using purified RNases H (Figure 2). These experiments are consistent with the notion that repeat expansions cause a corresponding increase in R-loops at the *FMR1* locus. Indeed, an upward trend in the *FMR1* S9.6-pulldown efficiency was observed for individuals with increasing CGG-repeat sizes (Figure 2*A*; Figure S3), despite the variation that exists between individuals within both control and premutation allele classes. Based on three control CGG subjects (2–3 independent replications per subject; n = 8 experiments) and four premutation subjects (2–4 independent replications per subject; n = 10 experiments), the fold enrichment in premutation (mean 9.0, SD 3.9, range 2.9–13.4) was significantly greater than for controls (mean 4.2, SD 2.4, range 1.6–8.6) (*P* = 0.0008; linear mixed-effects model; see: Materials and Methods).

Our DOX-inducible episomal *FMR1* system provides a more controlled isogenic platform to directly parse out the impact of repeat expansion and transcription frequency on R-loop formation. As expected, R-loop formation increased in direct response to increasing transcription (Figure 3*C*). CGG-repeat length at equivalent transcription levels, however, appeared to have little effect on R-loop frequency. Removing DOX resulted in a corresponding decrease in R-loops in the episomal *FMR1* 5′UTR (Figure 3*D*), demonstrating the plasticity of R-loop formation at a given locus, with formation driven by active transcription and dissolution catalyzed by native enzymes such as RNases H, RNA:DNA helicases, or DNA topoisomerases [21], [38].

R-loops at CGI promoters were recently implicated in mediating protection against DNA methylation and epigenetic silencing [18]. R-loops at *FMR1* likely contribute to the same function for alleles in the normal and premutation ranges, in which the 5′UTR remains essentially unmethylated. Additionally, R-loop formation, by fostering a more open chromatin environment [29], is possibly responsible for the increased transcription resulting from repeat expansion *in FMR1*, although there is no direct evidence for this suggestion. The *FMR1* CGI is unique among CGIs in that it undergoes hypermethylation and silencing for full mutation alleles (≥200 CGG repeats), which suggests that, above a certain genetically-encoded threshold, the protection force operating at *FMR1* may be overcome by an as-yet-undefined silencing mechanism (either at the DNA or histone level). This transition between protection and silencing regimes could in fact be mediated by the unusual nature of R-loops formed through expanded CGG repeats. We provide evidence here that the non-template strand of *FMR1* R-loops presents stretches that are refractory to bisulfite footprinting (Figure 4), indicative of possible hairpin-like structures, which is consistent with the well-documented propensity of trinucleotide CGG repeats to fold into higher-order structures [39], [40]. Such structural characteristics distinguish *FMR1* from other non-repetitive R-loop-forming CGI promoters [18], [19] and even from repetitive R-loop-forming sequences such as class-switch regions [26], [41].

**Figure 5:**
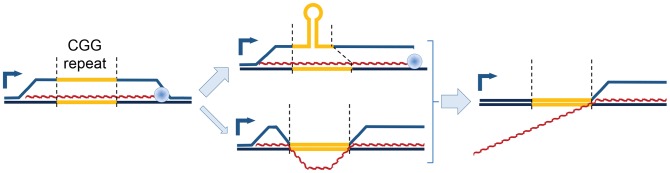
Model of proposed CGG-repeat effects on the *FMR1* R-loop. R-loops that span the *FMR1* CGG-repeat region (yellow) during transcription could adopt a hairpin structure within the displaced CGG-repeat strand, thus protecting the CGG-repeat region from bisulfite conversion while leaving both 5′ and 3′ flanking regions exposed; the CGG-repeat is known to form such structures readily *in vitro* [61]. An alternative structure, although less energetically feasible, would involve maintenance of R-loops flanking the CGG-repeat element, which has collapsed into a dsDNA structure again. Loss of the upstream R-loop region would explain the absence of bisulfite conversion in ∼25–50% of molecules (Figure 4). Red, nascent RNA transcript; 90° arrow, start of transcription; blue sphere, Pol II.

Our non-denaturing bisulfite footprinting data also show that regions of ssDNA often appear to be located downstream of the CGG repeats themselves, particularly for larger repeats (Figure 4). This pattern is unusual in that GC skew should favor R-loop initiation within the repeats. It is possible that hairpin formation on the displaced G-rich strand may cause collapse of the R-loop structure by imposing torsional stress on the RNA:DNA hybrid (Figure 5). Such hairpin-mediated interconversion between a “regular” R-loop and a “collapsed” R-loop would explain the patterns observed and would be compatible with the unique sequence characteristics of the region. Interestingly, the structural complexities observed at *FMR1* may have relevance to the transition from an active to a silenced state characteristic of fully expanded repeats. For instance, DNMT1, the most powerful DNA methyltransferase in human cell extracts, recognizes structured/hairpin DNA as a substrate for methylation [42]. This recognition could seed DNA methylation inside the repeats before spreading up- and down-stream over the rest of the UTR/promoter. Alternatively, collapse of the RNA:DNA hybrid inside the CGG repeats could potentially disrupt the protective effect of R-loops against DNA methylation, which has been observed at other CGI promoters [18]. Previous studies of *FMR1* hypermethylation have not mapped high-resolution methylation patterns inside the repeats themselves [43], [44], and thus would easily overlook this repeat-centric model.

In addition to changes in secondary structure, repeat expansion and increased transcription could result in R-loop-driven activation of the DNA damage response and genomic instability [22], [27], [28], [45]–[49]. R-loops in the inappropriate context or timing result in DNA breaks, as indicated by recruitment of γH2AX. In this regard, we have previously reported activation of the double-stranded-break repair pathway in this same episomal system, but only in highly transcribed expanded CGG repeats [36]. Even though elevated exogenous expression likely exaggerates this effect in the model system, γH2AX is observed in the characteristic intranuclear protein inclusions of post-mortem neurons in FXTAS patients [36]. In addition to potential involvement in FXTAS pathology, R-loop formation and/or DNA damage are responsible for genomic instability generally [22], [48], and at *FMR1* specifically [50], [51], including the eponymous fragile site [52]. In addition to toxicity and instability, DNA damage has been linked to aberrant DNA methylation [53]; increased R-loop formation and/or increased damage of a full mutation CGG R-loop could overcome the protective features of 5′UTR CGI R-loops and trigger the methylation and silencing characteristic of FXS.

We introduce here a previously unrecognized molecular feature of the *FMR1* gene that is influenced by expansion of the CGG-repeat element. R-loop formation is a normal and important feature of the *FMR1* promoter, but expansion of the CGG repeats, and the associated increase in transcription, results in increased formation of longer R-loops that are more prone to folding into complex secondary structures, which could trigger instability and hypermethylation associated with *FMR1*-repeat expansion. This discovery provides a novel area of inquiry for understanding the aberrant cellular responses to CGG-repeat expansion at *FMR1*, and at transcribed trinucleotide-repeat loci throughout the genome.

## Materials and Methods

### Cell Culture

Human dermal fibroblasts were originally cultured from skin biopsies acquired under an IRB-approved protocol, as previously described [54]. Cells were grown at 37°C, 5% CO_2_ in a 50∶50 mix of RPMI-1640, supplemented with 1× Amphotericin B (JR Scientific, Woodland, CA), 1× Penicillin-Streptomycin-Glutamine (Life Technologies, Carlsbad, CA), 1× MEM Non-Essential Amino Acids Solution (Life Technologies), and 10% Fetal Bovine Serum (JR Scientific) and AmnioMAX C100 media (Life Technologies). Fibroblasts were harvested at 80% confluency to avoid decreased transcription associated with contact inhibition.

SK-N-MC-rtTA cell lines harboring expanded CGG-repeat episomes were created, as previously described [36]. These cells were grown in DMEM (Life Technologies) +10% Tet-system-approved fetal bovine serum (Clontech, Mountain View, CA) and 1× Penicillin-streptomycin (Life Technologies) at 37°C, 5% CO_2_. DOX media was prepared from 1 mg/mL stock doxycycline hyclate (Sigma-Aldrich, St. Louis, MO) dissolved in sterile water. For the DOX washout, DOX media was aspirated and cells were washed once with DPBS (Life Technologies) before adding DOX-free media.

### Harvesting Nucleic Acids for DRIP

Adherent cells were trypsinized (0.25% trypsin; Life Technologies) for fibroblasts and 0.05% trypsin (JR Scientific) for SK-N-MC cells for 4 minutes at 37°C before quenching with an equal volume of media and pelleting at low speed (200 RCF). Cell pellets were washed with DPBS (Life Technologies) and divided for DRIP or RNA harvests. Cell pellets for RNA harvest were lysed in RLT buffer (Qiagen, Hilden, Germany) and frozen at −80°C before processing at a later date according to the RNeasy kit (Qiagen). Cell pellets for DRIP were resuspended in 4 mL of 10 mM Tris-HCl, 10 mM EDTA, 100 mM NaCl pH 8, lysed with 0.5% SDS, and digested with 400 units of Proteinase K (Thermo Fisher Scientific, Waltham, MA) at 37°C overnight. Cell lysates were then extracted once with 1 volume of equilibrated phenol pH 8 (USB, Cleveland, OH) and twice with 1 volume of chloroform (Sigma-Aldrich). DNA was precipitated with 1 volume of isopropanol and 300 mM sodium acetate, and was swirled out of solution with a glass shepherd's hook. The DNA pellet was washed twice by rinsing the hook with 400 µL of 70% ethanol, and was rehydrated in 10 mM Tris-HCl pH 8.

### DRIP

Harvested nucleic acids (∼50 µg) were digested using a restriction enzyme cocktail (20 units each of EcoRI, HindIII, BsrGI, XbaI) (New England Biolabs, Ipswich, MA; NEB) overnight at 37°C in 1× NEBuffer 2. Digests were cleaned by phenol and chloroform extraction followed by precipitation in isopropanol. The resulting fragmented DNA was pelleted at full speed (16,100× g) at 4°C and washed twice with 70% ethanol. Air-dried pellets were rehydrated in 10 mM Tris-HCl pH 7.5, 1 mM EDTA (TE).

We adapted the previously described DRIP protocol [18]. Six to eight µg of digested nucleic acids were diluted in 450 µL of TE, and 10 µL was reserved as input for qPCR. Fifty-two µL of 10× IP buffer was added for a final buffer concentration of 10 mM sodium phosphate, 140 mM sodium chloride, 0.05% Triton X-100, and 20 µL of S9.6 antibody (1 mg/ml; prepared from ascites, as previously described [18]). The samples were incubated with the antibody at 4°C for 2 hours. This incubation and all wash steps were performed on a rotisserie mixer. Forty µL of Protein A/G Agarose slurry (Pierce, Rockford, IL) was washed twice with 800 µL of 1× IP buffer for 5 minutes at room temperature. After adding agarose slurry to each sample, they were incubated for 2 hours at 4°C. Each DRIP was then washed three times with 700 µL 1× IP buffer for 10 minutes per wash at room temperature. After the final wash, the agarose slurry was resuspended in 250 µL of 1× IP buffer and incubated with 60 units of Proteinase K for 30 minutes at 50°C. Digested DRIP samples were then cleaned with phenol/chloroform extraction and isopropanol precipitation. Air-dried DRIP pellets were resuspended in 80 µL of 10 mM Tris-HCl pH 8.

We used 12 µL reactions with Sensi-FAST Lo-Rox 2× qPCR mix (Bioline, London, UK) to assay for genomic loci: *FMR1* (200 nM each) (F: TTGCCCCTTAGTTCCCTGAG; R:TCTTCCATCAGTGCAGACCA), *MYADM* (300 nM each) (F: CGTAGGTGCCCTAGTTGGAG; R: TCCATTCTCATTCCCAAACC), and *ZNF554* (300 nM each) (F: CGGGGAAAAGCCCTATAAAT; R: TCCACATTCACTGCATTCGT). For the episomal DRIP experiments, we assayed for EGFP (F: TCAAGATCCGCCACAACATC; R:TTCTCGTTGGGGTCTTTGCT) and the pCEP4 backbone (F:ATCCCCATCCCTACCGTCCA; R:CCCCATCCTCCGAACCATCC) using 5 µL of 1∶500 diluted template or 5 µL undiluted DRIP output (from 80 µL total). Reactions were incubated with the following program on a Viia 7 System (Life Technologies): 50°C 2 minutes, 95°C 10 minutes, 40 cycles of 95°C 15 seconds, 64°C 1 minute, followed by a melt curve: 95°C 15 seconds, 60°C 1 minute, 0.05°C/second to 95°C 15 seconds. For each DRIP sample, 5 µL of the output and 5 µL of diluted input (1∶100) were assayed in triplicate. Fold enrichment for a given locus (i.e., *FMR1* or *EGFP*) was calculated using the comparative Ct method [55], relative first to input and then to the appropriate reference (i.e., *ZNF554* or pCEP4 backbone).

Comparison of fold enrichment between premutation (4 subjects, 2–4 independent replications per subject; n = 10 experiments) and control (3 subjects, 2–3 independent replications per subject; n = 8 experiments) subjects (Figure 2) was based on a linear mixed-effects model to account for correlation between repeated measurements on the same subjects. The analysis was done using SAS version 9.3.

### Non-denaturing Sodium Bisulfite Mapping

Harvested nucleic acids (4–10 µg) were digested with HindIII (20 units, ∼5 hours at 37°C; NEB) and then treated with the sodium bisulfite conversion mix from the EZ-DNA Methylation Kit (Zymo Research, Irvine, CA) overnight at 37°C. Bisulfite-treated DNA was then desulphonated and cleaned according to kit protocol and was eluted in 10 µL 10 mM Tris-HCl pH 8.

Bisulfite-treated DNA was amplified using a method adapted for CGG-repeat amplification [56]. One to two µL of bisulfite-treated DNA was amplified in a 30 µL reaction with 0.5 mM dNTPs, 2.25 M betaine (Sigma), 333 nM of each primer in 1× buffer and 0.2 µL of enzyme mix from the Expand Long Template Kit (Roche, Basel, Switzerland). Enzyme and buffer were added after 8 minutes at 98°C, followed by an additional 2 minutes at 98°C, then 10 cycles at 97°C for 35 seconds, 64°C for 35 seconds, 68°C for 4 minutes, 25 cycles at 97°C for 35 seconds, 64°C for 35 seconds, 68°C for 4 minutes, plus a 20-second increment for each cycle, and a final extension at 68°C for 10 minutes. In order to successfully and cleanly amplify through the bisulfite-converted CGG repeats, we used two rounds of amplification with a nested primer set (first round: F:GAGGGAACAGCGTTGATCACGTG R: CACTTAACACCAATTTCAACCCTTCCCACC; second round: F: GGAACAGCGTTGATCACGTGACGTGGTTTC R: CTTCCCTCCCAACAACATCCCACCAAAC).

PCR-amplified DNA was sub-cloned using the Qiagen PCR Cloning Kit. Chemically competent *E. coli* Top10 cells (Life Technologies) were transformed by heat-shock with ligated plasmid, and were grown overnight at 37°C on LB agar plates with 100 mg/ml ampicillin selection. Picked colonies were grown in 4 mL LB broth with 100 mg/ml ampicillin at 30°C with 150 rpm shaking overnight; plasmid DNA was extracted using the Qiagen plasmid miniprep kit.

Plasmid DNA PCR clones were sequenced (Davis Sequencing, Davis, CA) with M13R or SP6 primers, depending on orientation of the PCR insert. Clean sequence clones were then aligned to an unconverted reference sequence with Clustal W2 [57] to score cytosine conversion events.

The full-length cDNA for human RNASEH1 (ATCC, Manassas, VA) was PCR-amplified, excluding the first 26 amino acids of the protein corresponding to the mitochondrial localization signal [58]. The amplified fragment was recloned in frame in a modified pMAL vector [59] to generate an MBP-RNASEH1 fusion protein. Protein expression was induced for 2 hours at 37°C in *E. coli* Rosetta cells grown in exponential phase in Terrific Broth. Cells were harvested and lysed with a microfluidizer in amylose buffer (10% glycerol, 25 mM Tris-HCl pH 7.5, 250 mM NaCl, 1 mM DTT, 0.5 mM EDTA supplemented with complete protease inhibitor cocktail; Roche), and the lysate was spun for 1 hour at 30,000× g. The supernatant was then applied to a 20 ml amylose column (NEB) equilibrated in 1× amylose buffer, after which the column was washed with 10 column volumes of binding buffer. The MBP-RNASEH1 protein was eluted in batch in binding buffer supplemented with 20 mM maltose. The protein was then dialyzed against Q buffer (20 mM Tris-HCl pH 7.5, 50 mM NaCl, 0.1 mM β-mercaptoethanol) overnight at 4°C and was applied to a pre-equilibrated 5 ml FastFlow Q column (GE Healthcare, Little Chalfont, UK) using an Akta FPLC system. The protein mostly flowed through. The flow-through was then re-applied to a Mono-Q column (GE Heathcare) to separate the protein from any contaminating nucleic-acid species. The flow-through was again collected, concentrated, and dialyzed against storage buffer (20 mM Tris-HCl pH 7.5, 50 mM NaCl, 1 mM DTT, 0.1 mM EDTA, 20% glycerol), then aliquoted and snap frozen in liquid nitrogen before storage at −80°C. The concentration of the preparation was calculated to be 17.7 µM (1.3 mg/ml) using an extinction coefficient of 112,710 M^−1^cm^−1^. The pMAR22 expression vector for the heterotrimeric RNASEH2 complex was a kind gift from Dr. Reijns; the complex was purified essentially as described [60]. The protein was stored as described for RNASEH1. The concentration of the preparation was calculated to be 15 µM (1.34 mg/ml) using an extinction coefficient of 81,050 M^−1^cm^−1^. Both preparations were ∼98% pure, as judged from Coomassie-stained SDS-PAGE gels, and gave expected sizes of either one single band for MBP-RNASEH1 or three equimolar bands for the RNASEH2 complex. Both preparations were devoid of detectable endo- or exonuclease activity after incubating 1 µl of undiluted protein with double-stranded circular or linear DNA substrates for 4 hours at 37°C (data not shown). Both preparations were highly active even under 10,000-fold diluted concentrations against artificial R-loop substrates prepared by *in vitro* transcription (data not shown).

## Supporting Information

Figure S1 R-loop formation at an endogenous positive genomic locus, *MYADM*. R-loop formation is reported after DRIP-qPCR for the *MYADM* CpG-island region. The data are acquired from genomic DNA samples obtained from dermal fibroblast samples cultured from seven different individuals. Enrichment is relative to input and normalized to a non-R-loop-forming genomic reference locus.
(PDF)

Figure S2 R-loop recovery after DRIP-qPCR is plotted as percentage of input for the target/GFP episome fragment (panel A; left) or the episome backbone (panel B; right) for three constructs (30 CGG, dark gray; 95 CGG, striped; Not *FMR1*, white). Error bars are SEM for 3 DRIP replicates.
(PDF)

Figure S3 R-loop recovery after DRIP-qPCR is shown for *FMR1* relative to the positive control *MYADM* using samples from dermal fibroblast cells cultured from seven individuals. A slightly higher recovery tends to be observed for individuals with longer repeats, suggesting that R-loop formation may be more efficient over longer repeats.
(PDF)
