# Supplementary figures and images for: Transcription-Associated R-Loop Formation across the Human FMR1 CGG-Repeat Region
Source: PLoS Genet. 2014 Apr 17;10(4):e1004294. doi: 10.1371/journal.pgen.1004294 (PMC3990486; doi:10.1371/journal.pgen.1004294)

# *MYADM*

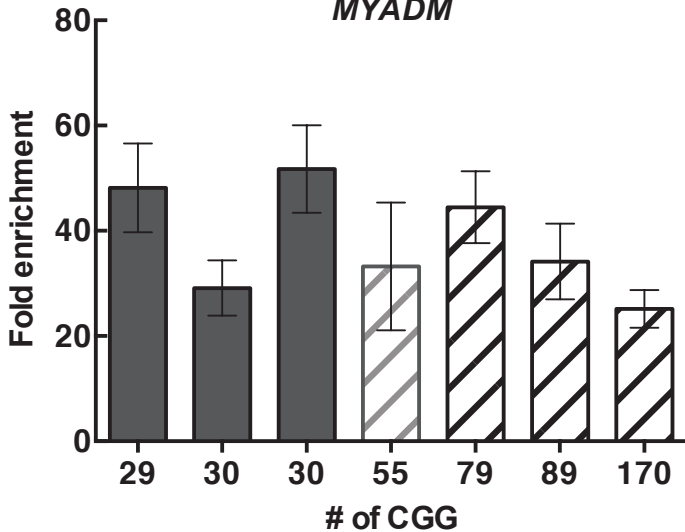

Supplement: Figure S1 — R-loop formation at an endogenous positive genomic locus, MYADM. R-loop formation is reported after DRIP-qPCR for the MYADM CpG-island region. The data are acquired from genomic DNA samples obtained from dermal fibroblast samples cultured from seven different individuals. Enrichment is relative to input and normalized to a non-R-loop-forming genomic reference locus. (PDF) [file pgen.1004294.s001.pdf]

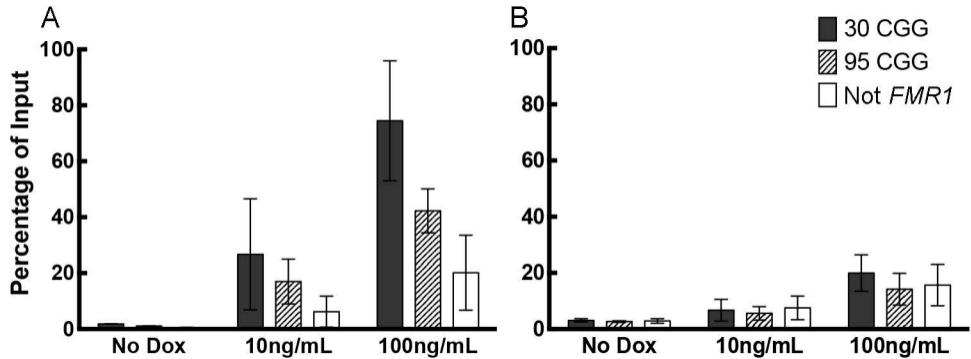

Supplement: Figure S2 — R-loop recovery after DRIP-qPCR is plotted as percentage of input for the target/GFP episome fragment (panel A; left) or the episome backbone (panel B; right) for three constructs (30 CGG, dark gray; 95 CGG, striped; Not FMR1, white). Error bars are SEM for 3 DRIP replicates. (PDF) [file pgen.1004294.s002.pdf]

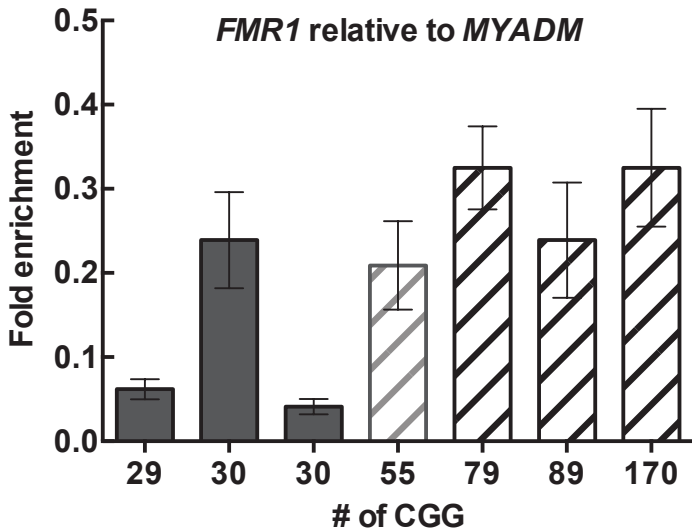

Supplement: Figure S3 — R-loop recovery after DRIP-qPCR is shown for FMR1 relative to the positive control MYADM using samples from dermal fibroblast cells cultured from seven individuals. A slightly higher recovery tends to be observed for individuals with longer repeats, suggesting that R-loop formation may be more efficient over longer repeats. (PDF) [file pgen.1004294.s003.pdf]
